# Personalized Medical Approach in Gastrointestinal Surgical Oncology: Current Trends and Future Perspectives

**DOI:** 10.3390/jpm15050175

**Published:** 2025-04-27

**Authors:** Dae Hoon Kim

**Affiliations:** 1 Department of Surgery, Chungbuk National University Hospital, Cheongju 28644, Republic of Korea; roadangel@hanmail.net; Tel.: +82-43-269-6293; 2 Department of Surgery, Chungbuk National University College of Medicine, Cheongju 28644, Republic of Korea

**Keywords:** personalized medicine, gastrointestinal surgical oncology, trend, future perspective, artificial intelligence, patient-centered care, genomic profiling

## Abstract

Advances in artificial intelligence (AI), multi-omic profiling, and sophisticated imaging technologies have significantly advanced personalized medicine in gastrointestinal surgical oncology. These technological innovations enable precise patient stratification, tailored surgical strategies, and individualized therapeutic approaches, thereby significantly enhancing clinical outcomes. Despite remarkable progress, challenges persist, including the standardization and integration of diverse data types, ethical concerns regarding patient privacy, and rigorous clinical validation of predictive models. Addressing these challenges requires establishing international standards for data interoperability, such as Fast Healthcare Interoperability Resources, and adopting advanced security methods, such as homomorphic encryption, to facilitate secure multi-institutional data sharing. Moreover, ensuring model transparency and explainability through techniques such as explainable AI is critical for fostering trust among clinicians and patients. The successful integration of these advanced technologies necessitates strong multidisciplinary collaboration among surgeons, radiologists, geneticists, pathologists, and oncologists. Ultimately, the continued development and effective implementation of these personalized medical strategies complemented by human expertise promise a transformative shift toward patient-centered care, improving long-term outcomes for patients with gastrointestinal cancer.

## 1. Introduction

Personalized medicine is far from a modern invention. Its origins date back to ancient times when physicians recognized that each patient requires a unique treatment approach. For example, Hippocratic medicine was developed to balance the four humors (blood, phlegm, yellow bile, and black bile) to tailor interventions to an individual’s natural constitution. Similarly, traditional healing practices in Egypt, India, and China relied on detailed observations of a patient’s symptoms and overall condition to provide customized care [1]. This tailored approach is also used in gastrointestinal surgical oncology, in which surgical treatment often involves not only primary tumor resection but also specialized lymphadenectomy [2,3]. This procedure targets lymph nodes that are predicted to harbor metastatic disease, acknowledging that each patient’s unique anatomical structure and cancer progression patterns require an individualized surgical strategy. This precision in determining the extent of lymph node removal underscores the inherent personalization of conventional surgical techniques [4]. In the modern era, the convergence of advanced diagnostic technologies, genomic profiling, and artificial intelligence (AI)-driven analytical tools has propelled personalized medicine to new heights. In gastrointestinal surgical oncology, the integration of AI with high-throughput genomic data enables clinicians to design highly individualized treatment plans that optimize surgical outcomes, reduce complications, and improve long-term survival. This seamless blend of ancient principles with cutting-edge technology underscores the enduring relevance and evolution of personalized medicine [5].

The emergence of AI, particularly deep learning, as a tool for personalized treatment is rooted in several transformative technological advancements. First, the widespread availability of vast, well-labeled datasets, combined with major improvements in computing power and cloud storage, has enabled deep learning algorithms to excel in processing complex medical data. This has led to breakthroughs in areas such as rapid and highly accurate image interpretation, allowing clinicians to detect subtle patterns that inform individualized diagnoses and treatment plans [6]. Moreover, AI is reshaping health systems by automating complex tasks, streamlining clinical workflows, and reducing the incidence of medical errors, ultimately enabling more precise and efficient patient care. Finally, AI is fostering a more engaged and proactive role in personalized medicine by providing patients with tools to analyze and interpret their health data. Although challenges such as bias, privacy, and transparency remain, the overall trajectory suggests that AI will continue to enhance the personalization of medical treatments, transforming patient care in profound ways [7].

This review aimed to explore the impact of personalized medicine in gastrointestinal surgical oncology by addressing three core areas. First, we review the current trends, including advances in genomic profiling, biomarker analysis, image-guided surgery, and tailored surgical planning, which set the stage for individualized patient care. Notably, image-guided surgery, which integrates advanced imaging modalities with AI and robotics, offers real-time visualization and precise intervention, thereby enhancing surgical accuracy and patient outcomes [8]. Second, we examine the contributions and applications of AI technology, particularly deep learning, which improves diagnostic accuracy, optimizes treatment strategies, streamlines clinical workflows, and bolsters image-guided interventions [7,9,10]. Finally, we discuss future perspectives and inherent limitations, such as data quality challenges, privacy concerns, and ethical issues that may influence the further integration of AI in patient care. Together, these discussions provide a comprehensive overview of the evolving landscape of personalized medicine in this field.

## 2. Current Trends in Personalized Medicine

### 2.1. Genomics and Biomarkers

Advances in high-throughput sequencing technologies, commonly known as next-generation sequencing (NGS), have fundamentally transformed tumor genomic profiling. These cutting-edge platforms enable simultaneous sequencing of millions of DNA fragments, allowing researchers to examine entire genomes, exomes, or focused gene panels in a single run. This unprecedented sequencing capacity not only accelerates the process but also enhances the depth of genomic insights [11,12]. One of the most significant advantages of NGS is its ability to detect the broad spectrum of genetic alterations that drive cancer progression. These include single nucleotide variations (point mutations), small insertions and deletions (indels), copy number variations, and complex structural rearrangements, such as translocations and gene fusions. By mapping these genetic variations, clinicians can obtain a comprehensive molecular profile of tumors, which is essential for identifying oncogenic drivers and tumor suppressor genes [11,13,14]. Precision medicine transforms cancer treatments by tailoring therapies to the unique molecular profile of each patient. By integrating multi-omic analyses such as genomics, proteomics, and metabolomics, clinicians can identify actionable biomarkers and target-specific genetic alterations, thereby maximizing therapeutic efficacy while minimizing adverse effects [11,12]. AI and machine learning (ML) further refine these strategies by analyzing extensive datasets of subgroups of patients based on tumor biology, thereby guiding the selection of targeted therapies and immunotherapies [5,7]. Non-invasive techniques, such as liquid biopsies, also enable the real-time monitoring of tumor dynamics and the early detection of resistance patterns, ensuring that complex genomic data are effectively integrated into clinical workflows [14]. Collectively, these advancements mark a transition from a generic treatment model toward a dynamic, patient-specific strategy that enhances clinical outcomes and quality of life.

Building on the wealth of genomic data and the identification of actionable biomarkers [11,12], the next critical step is integrating these insights into personalized treatment strategies. In gastrointestinal cancers, where tumor heterogeneity and complex mutation profiles are common, this integration becomes even more crucial [5,15]. Multi-omic approaches, which incorporate transcriptomics, proteomics, and metabolomics alongside genomic profiling, provide a comprehensive understanding of tumor biology [11,12,16], enabling clinicians to stratify patients more accurately and tailor treatment plans that target the specific molecular alterations present in each tumor [5].

AI and ML are at the forefront of this transformation [5,7,17]. These technologies facilitate the analysis of large-scale omic data and enhance clinical decision-making by predicting therapeutic responses [5,7]. For example, AI-driven algorithms can assess liquid biopsy results in real time, detect early signs of treatment resistance, and allow for timely therapeutic adjustments [14,18]. Moreover, by integrating data from imaging studies, clinical records, and multi-omic profiles, ML models can contribute to the development of predictive tools that can forecast patient outcomes with greater precision [7,10].

The seamless integration of high-throughput sequencing technologies with AI analytics represents a paradigm shift from conventional “one-size-fits-all” treatment approaches to dynamic patient-specific strategies. As these technologies continue to evolve, they are expected to enhance treatment efficacy, reduce toxicity, and significantly improve patient survival and overall quality of life [5,7].

### 2.2. Treatment Personalization

Building on the integration of genomic insights, AI is now actively used to design individualized treatment pathways in gastrointestinal surgical oncology. In the preoperative stage, advanced algorithms analyze diverse data, such as clinical records, imaging studies, and genomic profiles, to assess a patient’s overall condition and surgical risk. This comprehensive assessment refines surgical candidacy evaluation and aids in identifying subtle clinical indicators that may otherwise be overlooked [5,7,19]. Following this evaluation, decision support systems were used to recommend an optimal treatment strategy tailored to each patient. These systems integrate multimodal data to determine whether a patient benefits more from surgical intervention, targeted drug therapy, or a combination of both. For instance, in patients with an increased risk of postoperative complications, AI can suggest minimally invasive techniques or prehabilitation strategies to enhance recovery [5,7,19,20]. Conversely, when genomic profiling reveals actionable mutations, AI-driven models can prioritize targeted therapies that complement surgical management, ultimately enhancing treatment efficacy.

This paradigm shift from standardized protocols to personalized treatment plans demonstrates the transformative role of AI in gastrointestinal oncology. By aligning treatment strategies with each patient’s unique clinical profile, these approaches improve survival rates and enhance the patient’s quality of life, marking a significant milestone in the evolution of precision medicine.

#### 2.2.1. AI-Based Imaging

In the field of medical imaging, AI plays a critical role in improving the diagnostic accuracy and predicting tumor prognosis across multiple imaging modalities, including computed tomography (CT), magnetic resonance imaging (MRI), and pathology slide analysis. Advanced deep learning techniques, particularly convolutional neural networks (CNNs), enable the extraction of subtle features from high-resolution images that may be overlooked by traditional interpretation methods. This enhanced capability allows clinicians to assess key tumor characteristics, such as size, shape, and spatial relationships with surrounding tissues, with greater precision, thereby improving diagnostic accuracy and supporting informed treatment decisions [10,21].

#### 2.2.2. Hepatobiliary and Pancreatic (HBP) Imaging

*Liver:* AI has rapidly emerged as a transformative tool for HBP imaging, particularly for liver tumor detection and characterization. By leveraging deep learning algorithms and radiomic-based analysis, AI enables comprehensive evaluation of both focal and diffuse liver diseases [22,23]. These advancements have played a crucial role in the clinical workflow, including precise organ segmentation, volumetric analysis, lesion characterization, and prognostic modeling [24]. Accurate segmentation of the liver is essential for assessing its volume, shape, and function, particularly in the preoperative planning for patients with chronic liver disease or hepatic tumors. Rahman et al. [25] proposed a deep learning-based segmentation model, ResUNet, which integrates residual networks with the UNet architecture to enhance the segmentation accuracy in CT images. Their approach demonstrated a Dice similarity coefficient (DSC) of up to 99.2%, significantly improving segmentation precision while minimizing manual intervention. Trained on the three-dimensional (3D) Image Reconstruction for Comparison of Algorithm Database-1 (IRCADb-1) dataset, the model effectively delineates liver structures, making it a valuable tool in anatomically complex cases. In terms of lesion differentiation, Yasaka et al. [22] demonstrated that a CNN could distinguish benign from malignant liver lesions on contrast-enhanced CT scans, achieving an area under the curve (AUC) of 0.91, outperforming conventional radiological assessments. Similarly, Hamm et al. [24] developed a CNN-based classifier for liver tumor diagnosis based on multiphasic MRI that achieved an accuracy of 90.4% in differentiating hepatocellular carcinoma (HCC) from other liver malignancies, underscoring its potential for improving clinical decision-making. Further advances have been made in 3D liver segmentation. Lu et al. [26] introduced a CNN combined with graph-cut algorithms, achieving a mean DSC of 0.92, demonstrating robust segmentation performance in anatomically complex cases. Additionally, Vorontsov et al. [27] enhanced lesion segmentation by integrating liver segmentation as prior knowledge, increasing lesion detection accuracy from 78.3% to 85.6% and demonstrating its significant clinical impact (Table 1). These AI-driven approaches contribute to more precise and automated assessments in liver imaging, reducing interobserver variability, enhancing diagnostic confidence, and ultimately improving patient management and surgical planning.

Accurate segmentation of the biliary tree and tumor lesions is crucial for evaluating the tumor burden, assessing biliary obstruction, and guiding both surgical and interventional treatments. Kim et al. [28] introduced a deep learning-based reconstruction method that significantly reduced magnetic resonance cholangiopancreatography (MRCP) acquisition time by factors of 2.4 and 3.0 at 3T and 0.55T, respectively, while maintaining comparable image quality. In terms of tumor differentiation, deep learning models have shown promising results in distinguishing between malignant and benign biliary conditions. Haghbin et al. [29] developed a CNN that achieved an 88% accuracy in differentiating cholangiocarcinoma (CCA) from HCC using multiphase CT scans. These models improve differentiation in diagnostically challenging cases with overlapping imaging features.

*Biliary System:* AI has significantly enhanced prognostication and treatment response prediction in biliary tract cancers. Zhao et al. [30] developed a radiomic-based model utilizing preoperative gadolinium-enhanced MRI to predict early recurrence in patients with intrahepatic mass-forming cholangiocarcinoma, achieving an accuracy of 87.6%. Additionally, Jolissaint et al. [31] developed an ML radiomic model capable of predicting early liver recurrence following curative resection of intrahepatic CCA, with an AUC of 0.84. Furthermore, Viganò et al. [32] introduced a CT-based radiogenomic model that non-invasively predicts genetic alterations in intrahepatic CCA, achieving an AUC of 0.819 for *IDH1* mutations and 0.892 for *FGFR2* alterations. These AI-driven methodologies provide precise, automated assessments that reduce inter-observer variability and improve tumor staging, treatment selection, and surgical planning. Similarly, Yin et al. [33] used a CNN trained on contrast-enhanced CT (CECT) images to differentiate between gallbladder cancer (GBC) and benign gallbladder diseases, achieving an AUC of 0.81. Their approach further enhances the precise differentiation between benign and malignant conditions in the biliary system, particularly where radiological features overlap (Table 1).

*Pancreas:* Pancreatic cancer remains one of the most challenging malignancies to diagnose and manage because of its complex anatomical location, nonspecific early symptoms, and aggressive nature. Recently, AI has emerged as a transformative tool for pancreatic cancer imaging, offering novel solutions for early detection, tumor characterization, and treatment planning. Deep learning-based tumor detection using CECT and MRI is one of the most promising applications of AI for pancreatic cancer. Mukherjee et al. [34] developed a radiomic-based ML model that analyzed pre-diagnostic CT scans obtained 3–36 months before the clinical diagnosis. Their support vector machine classifier achieved an AUC of 0.98 and an accuracy of 92.2%, significantly outperforming two experienced radiologists in detecting pancreatic ductal adenocarcinoma (PDAC). Similarly, Chu et al. [35] developed a radiomic-based ML model for analyzing CECT scans, achieving an even higher accuracy of 99.2%, with a sensitivity of 100% and specificity of 98.5%. In addition to detection, radiomic-based AI models have proven valuable for tumor characterization and prognosis prediction in PDAC. Li et al. [36] developed a radiomic signature based on CECT imaging to predict lymph node metastasis and achieved an AUC of 0.944 in the primary cohort and 0.912 in the validation cohort. Similarly, Gu et al. [37] conducted a multicenter study validating a multiscale deep learning radiomic nomogram for predicting recurrence-free survival, with a concordance index (c-index) of 0.82 in the training set and 0.70 in an external test set.

AI has also been instrumental in assessing surgical resectability, an essential step in the management of pancreatic cancer. Bereska et al. [38] demonstrated that a deep learning-based model using CECT imaging accurately classified patients with PDAC into resectable, borderline-resectable, and unresectable categories with high precision. Furthermore, AI-driven segmentation models, as reported by Viviers et al. [39], significantly outperformed conventional radiologist-based assessments. Their deep learning-based model achieved a DSC of 0.89 in venous segmentation and 0.86 in arterial segmentation, thereby improving the accuracy and reliability of predicting surgical resectability in patients with PDAC.

Finally, AI-driven imaging analytics enable real-time quantitative assessments of treatment efficacy and facilitate timely therapeutic adjustments. Nasief et al. [40] developed an ML-based delta-radiomic model that utilized sequential CT scans obtained during chemoradiation therapy (CRT) to predict early treatment response in patients with pancreatic cancer. Their model, which incorporates texture features such as kurtosis, coarseness, and normalized entropy-to-standard-deviation difference, successfully distinguished responders from non-responders, achieving a cross-validated AUC of 0.94 and an accuracy of 90%. In a complementary study, Noda et al. [41] used a histogram analysis of iodine concentration and CT numbers derived from dual-energy CT imaging to quantitatively assess the chemotherapeutic response in patients with PDAC. Their analysis identified iodine concentration metrics (IC diagnostic factors) as significant indicators of chemotherapy response, with a sensitivity of 97.7%, specificity of 70.6%, and an AUC of 0.889, offering objective metrics to support timely clinical decisions (Table 1).

#### 2.2.3. Gastric Cancer

Medical imaging plays a pivotal role in the diagnosis, staging, and treatment planning for gastric cancer. The integration of AI, particularly deep learning and radiomics, has significantly enhanced imaging modalities, such as CT, MRI, and positron emission tomography-CT (PET-CT) [42]. These AI-driven technologies improve tumor characterization, predict treatment responses, and optimize surgical planning, ultimately leading to improved patient outcomes [42,43]. Traditional imaging methods for gastric cancer rely heavily on radiologists’ interpretations, which are inherently subjective and susceptible to inter-observer variability. In contrast, AI-based approaches, particularly those utilizing CNNs, have demonstrated superior performance in detecting early gastric cancer and differentiating between benign and malignant lesions. For instance, Hirasawa et al. [43] demonstrated that a deep learning model applied to endoscopic images achieved a diagnostic accuracy comparable to that of expert endoscopists. In addition to detection, CT-based radiomics has emerged as a valuable tool for improving preoperative staging. Wang et al. [44] developed a CT-based radiomic nomogram that accurately predicted lymph node metastasis in gastric cancer and outperformed conventional imaging assessments.

AI-assisted imaging has revolutionized surgical planning and intraoperative navigation in gastric cancer. Advanced CT-based AI models enable the precise 3D reconstruction of tumor margins, vascular structures, and lymphatic pathways, significantly enhancing preoperative planning and facilitating targeted surgical intervention. Matsuki et al. [45] demonstrated that preoperative 3D imaging using multidetector CT (MDCT) effectively assessed the vascular anatomy around the stomach before laparoscopy-assisted gastrectomy. Their findings emphasized the critical role of accurate anatomical visualization in integrating AI-based and augmented reality navigation systems into modern surgical practice.

Predicting treatment response remains a primary challenge in managing gastric cancer. AI-driven radiomic and deep learning models are powerful tools for assessing and forecasting the efficacy of neoadjuvant chemotherapy (NAC). A recent systematic review and meta-analysis by Adili et al. [46] evaluated the diagnostic accuracy of radiomic-based ML models in predicting NAC responses. Their analysis, which included data from 14 studies encompassing more than 3300 patients, found that radiomic models, particularly those utilizing CECT images, achieved a pooled c-index of approximately 0.76 in the validation cohorts. Moreover, integrating clinical data with radiomic features further improved the predictive performance, enhancing both sensitivity and specificity. These findings highlight the potential of radiomic-based approaches for the early identification of NAC responders and non-responders, facilitating timely therapeutic adjustments and enabling personalized treatment strategies for gastric cancer.

AI-driven radiomic analysis plays a crucial role in postoperative monitoring and recurrence prediction. A recent study by Huang et al. [47] developed a transfer learning-based radiomic nomogram to predict recurrence in patients with advanced gastric cancer. This study found that radiomic features extracted from pathological whole-slide images significantly outperformed conventional CT-based models in predicting recurrence. The transfer learning-based radiomic model demonstrated superior predictive performance, with an AUC of 0.8561–0.9195 in the validation cohorts. These results highlight the value of AI-based radiomics for tailored postoperative monitoring (Table 1).

#### 2.2.4. Colorectal Cancer (CRC)

Medical imaging plays a pivotal role in the diagnosis, staging, and treatment planning of CRC. The integration of AI, particularly deep learning and radiomics, has significantly enhanced conventional imaging modalities such as CT, MRI, and PET-CT [48,49]. These AI-driven technologies enable precise tumor characterization, refine prognostic assessments, and enhance surgical planning, ultimately advancing personalized CRC management. AI-powered imaging, particularly CNNs, has demonstrated superior accuracy in detecting CRCs compared with conventional radiologist interpretations. Yamada et al. [50] demonstrated that deep learning models applied to colonoscopic imaging achieved a diagnostic performance comparable to that of expert endoscopists, significantly reducing the rate of missed lesions. Additionally, CNN-based analyses of CT and MRI scans have enhanced the detection of tumor infiltration depth, lymph node metastasis, and distant spread [48,49].

Radiomic and ML models have significantly improved the accuracy of preoperative staging of CRC. By extracting quantitative imaging biomarkers from CT or MRI scans, these models allow for precise tumor characterization, enabling clinicians to stratify patients more effectively and tailor surgical or neoadjuvant treatment strategies based on individual tumor biology [17,44,48,49]. AI-assisted 3D reconstruction techniques have significantly improved surgical planning by providing detailed visualizations of tumor margins, vascular structures, and lymphatic pathways [5,20,44]. This technology enhances intraoperative navigation and facilitates minimally invasive approaches, thereby reducing surgical morbidity and improving patient outcomes [5,9].

AI-driven radiomic models play a crucial role in predicting treatment response to neoadjuvant CRT (nCRT) in rectal cancer. These models integrate multimodal imaging data to identify early responders and non-responders, enabling clinicians to devise timely adjustments to therapeutic regimens [48,49]. AI-based radiomic signatures derived from pretreatment imaging can accurately predict the pathological complete response (pCR) in patients with rectal cancer undergoing nCRT, supporting more effective treatment planning. A systematic review and meta-analysis reported that ML models achieved a pooled AUC of 0.91, with a sensitivity of 0.83 and specificity of 0.86 in predicting pCR to nCRT in patients with locally advanced rectal cancer [51]. Furthermore, Jia et al. [52] conducted a meta-analysis demonstrating that deep learning models utilizing MRI data outperformed traditional radiomic models in predictive accuracy (pooled AUC: 0.97 vs. 0.85), highlighting AI’s potential in improving treatment response prediction (Table 1).

**Table 1:** Summary of AI imaging models across GI cancers.

Cancer Type	Modality	AI Model/Method	Target Outcome	Performance Metrics	References
Liver	CT, MRI	ResUNet, CNN, Graph-cut CNN	Segmentation, Lesion differentiation, Prognosis	DSC up to 99.2%, AUC 0.91	[22,24,25,26,27]
Biliary	CECT, MRI	CNN, Radiomics, Radiogenomics	Lesion classification, Genetic mutation prediction, Recurrence risk	Accuracy 88%, AUC 0.81–0.892	[28,29,30,31,32,33]
Pancreas	CECT, MRI	SVM, CNN, Delta-Radiomics	Detection, Resectability, Prognosis, Treatment response	AUC up to 0.98, Accuracy 99.2%	[34,35,38,39,40,41]
Gastric	CT, MRI, PET-CT	Radiomics, Deep learning	Early detection, Lymph node metastasis, NAC response, Recurrence	C-index 0.76, AUC 0.8561–0.9195	[43,44,45,46,47]
Colorectal	CT, MRI, PET-CT	Radiomics, Deep learning CNN	Staging, pCR prediction, Recurrence detection	AUC 0.91–0.97, Sensitivity 0.83, Specificity 0.86	[50,51,52]

### 2.3. AI-Based Natural Language Processing (NLP) for Clinical Record Analysis

Recent advancements in AI have significantly enhanced medical data analysis, with NLP emerging as a key technology for extracting valuable insights from unstructured clinical data. Although AI-driven imaging analysis has revolutionized cancer diagnostics and prognosis prediction through CT, MRI, and pathology slide assessments, these methods often fail to capture patient-specific factors such as comorbidities, treatment history, and postoperative complications. To address these limitations, NLP complements AI imaging by extracting and analyzing unstructured textual data, including electronic health records (EHRs), pathology reports, surgical notes, and documentation of clinical progress [7,19,48]. NLP-based AI models process vast amounts of clinical text, identify patterns, and extract critical medical insights that can inform personalized treatment strategies. For instance, deep learning-based NLP algorithms can automatically classify pathological findings, predict treatment responses, and assess disease progression through entity recognition and sentiment analysis [48,49,53]. In gastrointestinal surgical oncology, these models enhance risk stratification, refine prognostic assessments, and optimize therapeutic decision making by integrating real-world patient data with traditional imaging biomarkers.

A primary advantage of NLP in oncology is its ability to automate the real-time extraction of key clinical data from EHRs, thereby improving the efficiency and accuracy of medical decision making. ML algorithms can systematically analyze surgical reports and intraoperative findings to predict postoperative morbidity and recurrence risk. Additionally, NLP enables the seamless integration of real-world evidence by incorporating insights from the latest clinical guidelines, medical literature, and multi-institutional datasets, ensuring that treatment recommendations align with the most recent advancements in cancer care [7,19]. The synergy between NLP-driven text analysis and AI-based imaging forms the foundation of multimodal AI analysis, which is a cutting-edge approach that combines structured imaging data (e.g., tumor dimensions and radiomic features) with unstructured clinical narratives to enhance predictive modeling and decision support systems. For instance, integrating CT-derived radiomic biomarkers with NLP-extracted pathological descriptions improves recurrence risk prediction in gastric cancers and CRCs [5,49]. Similarly, in pancreatic cancer, the combined analysis of radiological reports, histopathological findings, and real-time surgical notes enhances patient stratification and guides postoperative surveillance strategies [5,7,37,48]. As AI-driven personalized medicine continues to evolve, NLP is expected to play a critical role in predictive analytics, clinical workflow automation, and individualized treatment planning. However, several challenges remain, including the need for standardized data across diverse healthcare systems, the mitigation of biases in NLP model training, and ethical considerations related to patient privacy [7,19,49,54]. Despite these challenges, the integration of NLP and imaging-based AI represents a paradigm shift toward a comprehensive data-driven approach for gastrointestinal cancer management, ultimately improving surgical outcomes and long-term patient survival.

#### 2.3.1. HBP Cancer

*Liver*: Recent advances in liver cancer management have integrated AI-driven imaging analysis with NLP-based genomic profiling, thereby significantly enhancing precision medicine. AI-assisted radiomics has demonstrated a superior performance in detecting molecular subtypes by extracting quantitative imaging features from CT scans. For instance, a study on CT-based radiogenomics reported high predictive performance for actionable mutations, such as *FGFR2* and *IDH1* alterations, achieving an AUC of approximately 0.89 for *FGFR2* mutation prediction [32]. Concurrently, deep learning models leveraging EHRs have been employed to predict critical clinical outcomes. NLP techniques applied to unstructured clinical narratives from pathology reports and EHR data have shown promising results in forecasting postoperative complications and liver failure. Rajkomar et al. [53] demonstrated that deep learning using EHR data can achieve robust predictions across various clinical scenarios, highlighting the potential of these methods to complement imaging-based genomic profiling. These findings support a new approach in precision oncology through the integration of imaging and clinical text analysis (Table 2).

*Biliary System*: Biliary tract cancers, including CCA and GBC, present significant diagnostic and therapeutic challenges owing to their aggressive nature and late stage presentation. The integration of AI-driven imaging analysis and NLP-based clinical data extraction has transformed the management of these malignancies by enhancing early detection, improving prognostication, and refining treatment strategies. Deep learning-based imaging analysis for lesion characterization and early diagnosis is one of the most promising applications of AI in biliary cancer. Radiomic-based AI models have demonstrated superior accuracy in differentiating malignant from benign biliary lesions using CECT and MRI scans. Haghbin et al. [29] developed a CNN model that achieved 88% accuracy in distinguishing CCA from HCC on multiphase CT imaging, significantly outperforming conventional radiological assessments. Similarly, Yin et al. [33] applied a CNN to CECT images to differentiate GBC from benign gallbladder diseases, achieving an AUC of 0.81. These AI-driven models enhance diagnostic precision and provide quantitative imaging biomarkers that can predict tumor aggressiveness and patient prognosis.

In addition to diagnosis, AI has shown promise in biliary cancer prognosis and treatment response prediction. Radiomic-based prediction models that use preoperative gadolinium-enhanced MRI have been developed to assess the risk of early recurrence in patients with intrahepatic CCA. Zhao et al. [30] demonstrated that this AI-driven model achieved an accuracy of 87.6% in predicting early recurrence, enabling clinicians to refine post-treatment surveillance and therapeutic planning. Additionally, Jolissaint et al. [31] introduced an ML-based radiomic model capable of forecasting early liver recurrence following the surgical resection of intrahepatic CCA, with an AUC of 0.84, highlighting its potential for postoperative risk stratification. Furthermore, AI-driven radiogenomic models have been employed to non-invasively predict key genetic alterations in biliary cancers. Viganò et al. [32] developed a CT-based radiogenomic model that identified *IDH1* mutations with an AUC of 0.819 and *FGFR2* alterations with an AUC of 0.892, providing valuable molecular insights that can guide the selection of targeted therapies.

The integration of NLP with AI-based imaging has further refined biliary cancer management. NLP algorithms applied to EHRs, pathology reports, and surgical notes can be used to extract crucial clinical information and improve risk stratification and treatment planning. For instance, deep learning-based NLP models have been utilized to identify high-risk patients with biliary cancer, automate pathology classification, and predict postoperative complications [55,56]. Ye et al. [55] demonstrated that NLP-driven predictive modeling could optimize tailored treatment strategies by analyzing unstructured clinical texts along with structured imaging data. By combining NLP with AI-based imaging, biliary cancer care is enhanced through improved risk assessment and treatment personalization (Table 2).

*Pancreas:* Pancreatic cancer remains one of the most challenging malignancies because of its late-stage diagnosis, aggressive progression, and limited therapeutic options. Advances in AI have significantly improved tumor characterization, resectability prediction, and treatment monitoring. In particular, the integration of radiomic- and NLP-based pathological analyses has enhanced subtype differentiation and personalized treatment. Radiomic-driven tumor classification has demonstrated superior accuracy in distinguishing PDAC from benign conditions, such as mass-forming pancreatitis. A deep learning radiomic model analyzing multiphase CECT and MRI scans achieved an AUC of 0.98, significantly outperforming expert radiologists in early PDAC detection [34]. Assessing resectability remains a critical challenge in pancreatic cancer management. Traditional imaging-based evaluations of vascular invasion and the tumor interface with the superior mesenteric artery and celiac axis are often subjective. AI-driven segmentation models provide quantitative real-time assessments to improve surgical planning. A deep learning model predicting vascular involvement achieved an AUC of 0.94, offering higher precision than conventional resectability criteria [38].

Moreover, the integration of AI-driven vascular segmentation with NLP-extracted surgical records further refines surgical strategies and guides decisions regarding vascular resection, NAC, and alternative treatment options [55,56]. In particular, NLP enhances traditional AI-based imaging analysis by incorporating crucial patient-specific data, such as prior treatment history, intraoperative findings, and postoperative complications, enabling more precise and personalized treatment decisions. In addition to preoperative decision-making, AI is transforming treatment response prediction and post-surgical monitoring. Delta-radiomic models that track sequential imaging during NAC or CRT can predict the tumor response and recurrence risk with high accuracy. A recent study demonstrated that radiomic-based predictive models identified early responders to NAC with 90% accuracy [40]. Currently, NLP-based research is prevalent in gastric and colorectal cancers, whereas studies specifically focusing on pancreatic cancer remain limited [57]. AI-driven vascular imaging and NLP-extracted surgical data improve treatment planning and monitoring in pancreatic cancer. Broader applications are needed, particularly for real-time treatment adjustments (Table 2).

#### 2.3.2. Gastric Cancer

A primary application of AI-integrated NLP in gastric cancer management is its ability to extract and analyze real-world clinical data from EHRs, pathology reports, and surgical notes. These AI-powered models extract crucial histopathological markers and molecular subtypes to predict postoperative complications [57]. NLP algorithms trained on large-scale datasets improve preoperative risk stratification, enabling the optimal selection of surgical candidates and neoadjuvant therapy responders [56]. Additionally, NLP-based models can automate pathology report analysis, enabling the rapid identification of high-risk features such as perineural invasion, lymphovascular invasion, and microsatellite instability (MSI) status, all of which have significant implications for treatment decisions [55]. By integrating these extracted features with radiomic and genomic data, AI models can provide a comprehensive multimodal assessment of tumor biology [49].

NAC has become a standard treatment strategy for locally advanced gastric cancer; however, response rates vary significantly among patients. AI-powered NLP models enhance treatment response predictions by analyzing both structured and unstructured clinical data. For instance, ML algorithms trained on pretreatment CT scans, histopathology reports, and genomic biomarkers predict chemotherapy responses with high accuracy. A meta-analysis by Adili et al. [46] found that radiomic-based AI models predict NAC responses more accurately than conventional imaging-based methods, with a pooled c-index of 0.76. Additionally, NLP-extracted clinical insights, when integrated with radiomic-based features, further refine predictive models, offering real-time guidance for modifying treatment strategies and optimizing chemotherapy regimens.

Early recurrence following curative gastrectomy remains a major clinical challenge. AI-integrated NLP models have been increasingly used to assess recurrence risk based on surgical notes, pathological findings, and genomic alterations. Huang et al. [47] developed a transfer learning radiomic nomogram that integrated radiomic features extracted from postoperative CT images with transfer learning signatures derived from whole-slide histopathological images. This integrated model significantly improved the prediction of postoperative recurrence of advanced gastric cancer. In their multicenter retrospective study involving 431 cases, the nomogram achieved an AUC of 0.9643 (95% confidence interval [CI], 0.9349–0.9936) in the training cohort and 0.8561 (95% CI, 0.7571–0.9552) to 0.9195 (95% CI, 0.8670–0.9721) in the validation cohort. These results highlighted the potential of AI in personalized postoperative surveillance. Beyond static risk assessment, AI-driven NLP algorithms enable continuous monitoring of longitudinal patient data and the detection of signs of recurrence earlier than conventional follow-up methods [57]. This approach allows for timely intervention and improves patient outcomes.

Despite these advancements, several challenges remain in the clinical implementation of AI and NLP in the management of gastric cancer. Data heterogeneity, variations in radiological and endoscopic interpretations, and the limited availability of large, high-quality labeled datasets pose significant obstacles [56]. Furthermore, algorithmic bias, model transparency, and privacy concerns have necessitated ongoing efforts to enhance the trustworthiness of AI-powered oncological applications [53]. However, as AI and NLP technologies continue to advance, despite remaining challenges, integrating AI and NLP is expected to advance precision care in gastric cancer and improve patient outcomes (Table 2).

#### 2.3.3. CRC

CRC remains the leading cause of cancer-related mortality worldwide, with significant variations in tumor biology and treatment responses. AI-integrated NLP transforms CRC management by extracting clinically relevant insights from EHRs, pathology reports, and surgical notes [57]. These AI-powered models improve risk stratification, recurrence prediction, and treatment selection by combining textual data analysis with imaging-based radiomic and genomic profiling [55]. A key application of NLP in CRC is the automated extraction of histopathological features and tumor subtyping. Deep learning-based NLP models facilitate the identification of high-risk pathological markers, including perineural invasion, lymphovascular invasion, and mismatch repair status, which are crucial for guiding treatment decisions [55,58]. Furthermore, ML algorithms trained on large-scale datasets enhance preoperative risk assessment, aiding the selection of patients who may benefit from neoadjuvant therapy before surgical intervention [52,59].

nCRT is the standard treatment for locally advanced rectal cancer; however, predicting the patient response remains a significant challenge. Multimodal AI frameworks that integrate deep learning-based imaging analysis with NLP-driven extraction of clinical, histopathological, and molecular features have shown promising potential for improving response prediction. For instance, Jia et al. [52] conducted a meta-analysis demonstrating that deep learning-based MRI analysis significantly outperformed conventional radiomic models in predicting pCR, with pooled AUC values of 0.97 vs. 0.85. Additionally, NLP-driven predictive modeling applied to EHRs and pathology reports has further refined the response stratification, enabling personalized treatment adjustments for nCRT [55,59].

The early detection of recurrence after curative resection is critical for patients with CRC because recurrence significantly affects long-term survival. AI-integrated NLP models have been used to assess recurrence risk by analyzing EHR-derived clinical data, pathological findings, and genomic alterations [57]. A recent study demonstrated that NLP-assisted risk modeling, when combined with radiomic-driven imaging analysis, significantly improved early recurrence detection compared with conventional follow-up strategies [55,60]. Furthermore, deep learning algorithms can continuously monitor longitudinal patient data, allowing for the real-time surveillance of post-treatment disease progression and recurrence risk assessment [53,60]. These advancements highlight the potential of AI-powered NLP for refining CRC management by providing a more precise and data-driven approach for treatment personalization and postoperative monitoring (Table 2).

**Table 2:** NLP-integrated AI models in GI oncology.

Cancer Type	AI/NLP Application	Best Performance Metrics	References
Liver	Postoperative complication prediction using EHR + radiogenomics	FGFR2 AUC ≈ 0.89 [32], Complication prediction	[32,53]
Biliary	Lesion classification, recurrence prediction, genetic mutation detection, NLP-based risk stratification	CCA recurrence AUC = 0.84, IDH1 AUC = 0.819, FGFR2 AUC = 0.892, NLP risk modeling	[29,30,31,32,33,55]
Pancreas	Subtype classification, vascular involvement prediction, NLP for surgical strategy optimization	PDAC detection AUC = 0.98, Vascular resectability AUC = 0.94, NLP surgical notes integration	[34,38,55,56]
Gastric	Pre/postoperative risk prediction, NAC response prediction, pathology feature extraction via NLP	Recurrence AUC = 0.9643, NAC response c-index = 0.76, Pathology NLP	[46,47,55]
Colorectal	nCRT response prediction, recurrence risk modeling, continuous post-treatment monitoring	pCR prediction AUC = 0.97, recurrence prediction, NLP-driven risk modeling	[52,55,57,60]

## 3. Role of Personalized Medicine in Gastrointestinal Surgical Oncology

Personalized medicine in gastrointestinal surgical oncology represents a paradigm shift from standardized treatment protocols to tailored therapeutic strategies that account for individual patient characteristics. Unlike conventional approaches that rely on generalized clinical guidelines [2,3], personalized medicine leverages patient-specific data such as genomic profiling [11,12], molecular biomarkers [14], and individualized risk assessments to optimize both surgical and adjuvant treatment plans. Advances in high-throughput genomic sequencing, proteomics, and metabolomics have enhanced our understanding of tumor biology [11,16], enabling clinicians to stratify patients based on their unique molecular signatures. This precision facilitates targeted interventions that minimize unnecessary treatments and enhance surgical outcomes [5]. Moreover, the integration of multimodal data, including histopathological findings, advanced imaging modalities [8], and comprehensive clinical parameters, has refined the decision-making process in gastrointestinal oncology, heralding a new era of personalized surgical care [5,7].

### 3.1. Genomic Profiling and Biomarker-Driven Surgery

One of the most transformative aspects of personalized medicine in surgical oncology is the application of genomic profiling. High-throughput sequencing technologies, such as NGS, have identified key genetic mutations and molecular aberrations that influence tumor behavior [11,12]. This has led to the development of biomarker-driven surgical strategies in which the extent of resection, lymphadenectomy, and neoadjuvant therapy is tailored to the patient’s genetic landscape [11,12,14]. For instance, in gastric cancer, human epidermal growth factor receptor 2 (HER2) status significantly influences treatment planning. Patients with HER2-positive tumors benefit from targeted therapies such as trastuzumab in conjunction with surgery, whereas HER2-negative patients may follow a different therapeutic pathway [15]. Similarly, in CRCs, MSI status is a critical determinant of immunotherapy response, guiding perioperative treatment decisions [14].

In hepatic and pancreatic malignancies, precision surgery is increasingly being guided by genomic markers that predict tumor recurrence and chemotherapy responsiveness [32]. Patients with intrahepatic CCA with *FGFR2* fusions or *IDH1* mutations may receive targeted preoperative therapies to enhance surgical outcomes [32]. Moreover, these advances not only refine the surgical approach but also inform adjuvant therapy choices, ultimately contributing to improved long-term survival. Such biomarker-driven decision making exemplifies the role of personalized medicine in refining oncological surgery [5].

### 3.2. Individualized Surgical Strategies in Gastrointestinal Cancers

#### 3.2.1. HBP Surgery

*Liver*: Personalized approaches in hepatic surgery have evolved significantly through precise preoperative assessments, particularly in evaluating future liver remnants (FLRs). Advanced volumetric imaging techniques combined with liver function tests facilitate tailored hepatic resection, thereby reducing the risk of post-hepatectomy liver failure. Recent advancements have incorporated AI-driven liver segmentation methods to improve FLR evaluation by accurately delineating healthy and compromised hepatic tissues. For example, Rahman et al. introduced the ResUNet model, which significantly improved segmentation precision with a DSC of 99.2% by integrating residual networks with a UNet architecture [25]. Additionally, AI-assisted radiomic analyses have refined tumor characterization, improved differentiation between benign and malignant lesions, and enabled more precise surgical targeting [22,24]. These integrated technologies have marked a shift toward personalized surgical care in hepatobiliary oncology.

*Biliary System:* In biliary tract cancers, where accurate diagnosis and staging are challenging, AI applications have advanced personalized treatment planning. AI-based deep learning algorithms offer precise differentiation between benign and malignant biliary lesions, thereby significantly improving the diagnostic accuracy. For instance, Haghbin et al. [29] reported that CNN models achieved an accuracy of approximately 88% in distinguishing CCA from HCC using multiphase CT scans. Moreover, recent radiogenomic approaches have utilized CT imaging to noninvasively predict critical genetic mutations, such as *IDH1* and *FGFR2*, with high accuracy, thus guiding targeted preoperative therapies and refining surgical decision-making [32]. In addition, AI-driven predictive models using preoperative MRI have demonstrated robust performance in forecasting early post-resection recurrence, thereby facilitating personalized surveillance strategies and adjuvant therapy planning [30,31]. These advancements underscore the critical role of integrating advanced imaging and genomic profiling with AI-driven analytics to further refine and personalize biliary cancer surgery, ultimately enhancing patient outcomes.

*Pancreas:* Given the aggressive nature and complex anatomical relationships of pancreatic cancer, personalized surgical strategies are critical. Recent advancements in AI-driven imaging have enhanced tumor detectability, resectability assessment, and intraoperative planning. Bereska et al. demonstrated that AI-based vascular involvement assessments using CECT imaging can accurately categorize tumors as resectable, borderline-resectable, or unresectable, offering superior accuracy compared with traditional methods [38]. Furthermore, deep learning-based segmentation algorithms have markedly improved the precision of vascular delineation, which is essential for determining safe surgical margins and guiding vascular resection during pancreaticoduodenectomy [39]. When integrated with conventional preoperative volumetric and functional evaluations, these innovations provide comprehensive and individualized surgical strategies aimed at reducing operative morbidity and enhancing patient survival [34,36,37].

#### 3.2.2. Gastric Cancer

Moreover, molecular profiling further refines the approach to lymphadenectomy by identifying patients with gastric cancer at a high risk of aggressive metastatic patterns. Comprehensive genomic characterization, including HER2 amplification, MSI, and *TP53* mutation status, has become an integral part of treatment stratification. For example, patients with HER2-positive or MSI-high tumors may benefit from tailored surgical approaches combined with targeted therapies or immunotherapies [14,15]. Additionally, radiomic-based imaging and ML-driven analysis provide a more precise method for lymph node metastasis prediction, thereby enhancing surgical decision-making. Wang et al. [44] developed a radiomic-based nomogram using preoperative CT imaging that significantly outperformed traditional clinical methods in predicting lymph node metastasis, thereby providing surgeons with robust data to guide surgical planning. Similarly, recent systematic analyses have indicated that integrating CT-based radiomic signatures into predictive models enhances NAC response assessment and overall patient stratification [46,49]. Sentinel lymph node mapping using dye or radioisotope techniques has been validated as a minimally invasive method for accurately identifying patients who could benefit from less extensive lymphadenectomy, thereby reducing postoperative complications without compromising oncological outcomes [4]. The convergence of advanced imaging, ML-based risk stratification, and genomic profiling represents a pivotal step toward truly personalized surgical strategies in gastric cancer, optimizing lymphadenectomy precision, and improving patient outcomes [5,7,42,47,49].

#### 3.2.3. CRC

In CRC, personalized surgical planning increasingly emphasizes the tailored determination of the extent of resection based on meticulous preoperative staging, patient-specific tumor characteristics, and predictive biomarkers. The optimal surgical extent, ranging from local excision to extended radical resection, depends on multiple factors, including tumor stage, precise anatomical location, involvement of adjacent structures, and lymphatic spread. Advancements in imaging technologies, such as high-resolution MRI and PET-CT, combined with radiomics have significantly enhanced the accuracy of preoperative staging, particularly in assessing tumor invasion depth and regional lymph node metastasis. A recent systematic review highlighted the role of radiomic analysis in predicting lymph node metastasis in CRC, further supporting the integration of imaging biomarkers into personalized surgical planning [61]. Moreover, the integration of MRI-based radiomic analysis has demonstrated high predictive accuracy in assessing the pCR following nCRT in rectal cancer, informing decisions regarding organ preservation versus radical resection [51,52]. Recent AI-driven histopathological analyses have further enhanced recurrence risk prediction in patients with CRC, offering new insights into long-term surgical outcomes [60,62].

In colon cancer, the tailored approach extends to deciding between segmental colectomy and extended hemicolectomy based on detailed assessments of the vascular anatomy and lymphatic drainage patterns. Recent studies have advocated individualized lymphadenectomy strategies, integrating sentinel node mapping techniques and molecular profiling, to identify patients who may benefit from extended lymph node dissections, thereby optimizing oncological outcomes while reducing unnecessary surgical morbidity [5,59,63]. A systematic review and meta-analysis of lymphatic mapping in colon cancer demonstrated how injection timing and tracing agents influence lymph node detection, supporting the refinement of sentinel node mapping strategies for more precise lymphadenectomy planning [63]. ML models that analyze postoperative outcomes have further refined these strategies by predicting recurrence risks and guiding postoperative surveillance [59,62]. These personalized surgical strategies for CRC underscore the evolution from generalized surgical guidelines towards nuanced, patient-specific approaches, ultimately aiming to enhance clinical outcomes, reduce postoperative complications, and improve overall survival [5,7,49].

### 3.3. Functional and Physiological Considerations

#### 3.3.1. Nutritional Status

In the era of personalized medicine, the optimization of nutritional status has evolved from applying standardized diet protocols to designing individualized nutritional interventions based on a patient’s unique metabolic, genomic, and inflammatory profiles. In gastrointestinal surgical oncology, where malnutrition frequently complicates treatment outcomes, tailored nutritional strategies are becoming increasingly vital. Advances in nutrigenomics and metabolomics have enabled clinicians to identify specific nutritional deficiencies and metabolic derangements contributing to immune dysfunction and impaired wound healing. For instance, preoperative assessments that integrate multi-omic data can determine which patients are most likely to benefit from targeted immunonutrition regimens. These personalized interventions may involve customized enteral feeding protocols enriched with bioactive nutrients such as omega-3 fatty acids, arginine, and nucleotides, each selected and dosed based on individual patient profiles, to enhance immune competence, reduce postoperative infections, and accelerate recovery [16,64,65]. By moving beyond the one-size-fits-all nutrition support, this approach exemplifies how the integration of precision diagnostics with tailored therapeutic strategies can significantly improve surgical outcomes and quality of life for patients with gastrointestinal cancers.

#### 3.3.2. Frailty and Comorbidity Assessment

In personalized surgical planning, a comprehensive evaluation of frailty and comorbidities has become a critical element in optimizing perioperative care for high-risk patients with gastrointestinal cancer. Modern protocols integrate standardized frailty indices, cardiopulmonary function tests, and enhanced recovery pathways to tailor surgical strategies based on the unique physiological reserve of each patient. This individualized approach refines risk stratification and informs decisions regarding the suitability of minimally invasive techniques. Recent evidence suggests that incorporating detailed frailty assessments into the preoperative workup significantly improves the prediction of postoperative complications and aids in selecting patients who are most likely to benefit from less invasive procedures [66,67]. By aligning surgical interventions with individualized health profiles, these strategies contribute to reduced morbidity, shorter recovery times, and improved overall surgical outcomes.

#### 3.3.3. Neoadjuvant Therapy Selection

Advances in molecular profiling have revolutionized the selection of neoadjuvant therapies in gastrointestinal oncology. Currently, CRT regimens are increasingly tailored using predictive biomarkers, ranging from genetic mutations to protein expression profiles, to identify patients most likely to benefit from intensive preoperative treatments [46,52]. This biomarker-driven approach ensures that patients who are unlikely to respond are spared the potential toxicities of unnecessary CRT, whereas those with favorable molecular signatures receive targeted treatment. Moreover, incorporating detailed biomarker profiling into treatment planning has been associated with higher rates of tumor downstaging, which not only facilitates more effective surgical resection but also contributes to improved overall outcomes [14]. By dynamically adjusting treatment based on molecular insights, this personalized strategy enhances the precision of neoadjuvant therapy, ultimately optimizing both the therapeutic response and surgical success.

### 3.4. Integration of Advanced Imaging and Real-Time Decision Support

Recent advances in imaging modalities and real-time data analytics have substantially enhanced personalized surgical planning for various gastrointestinal malignancies. Multiparametric imaging, including functional MRI, PET-CT, and radiomics-based analyses, provides quantitative insights into tumor biology, thereby enabling precise characterization and risk stratification [8,42,68]. The seamless integration of these imaging tools with AI-driven decision support systems enables surgeons to tailor operative strategies according to the unique anatomical and pathological profiles of each patient.

#### 3.4.1. HBP Surgery

*Liver*: Preoperative planning in hepatic surgery now leverages high-resolution volumetric imaging along with AI-assisted liver segmentation. Detailed 3D reconstructions delineate the hepatic anatomy, including vascular networks and parenchymal volumes, allowing for accurate simulation of resection scenarios and precise prediction of postoperative liver function. This integrated approach minimizes the risk of post-hepatectomy liver failure while optimizing the resection margins [32,45].

*Biliary System:* Accurate delineation of the biliary tree is critical for planning biliary tract cancer resection. Advanced imaging modalities such as MRCP and CECT, coupled with AI-enhanced radiomic techniques, enable detailed visualization of the biliary anatomy and improve differentiation between benign and malignant lesions. These innovations facilitate the precise mapping of the biliary system and surrounding vasculature, thereby refining surgical planning and reducing perioperative risks [29,32].

*Pancreas:* Pancreatic tumors pose significant challenges due to their anatomical complexity and aggressive nature. Advanced imaging modalities such as diffusion-weighted MRI and CT radiomics are pivotal in assessing tumor resectability by quantifying features indicative of vascular involvement and tissue invasion. Emerging AI algorithms further enhance these assessments by accurately predicting critical parameters, such as vascular encasement, thereby guiding surgical decision-making and reducing the need for unnecessary exploratory procedures [38,69].

#### 3.4.2. Gastric Cancer

In gastric cancer, the integration of advanced imaging techniques involves transforming preoperative evaluation and surgical planning. MDCT, PET-CT, and high-definition endoscopic imaging augmented by AI-powered radiomic analyses provide detailed maps of tumor margins, depth of invasion, and vascular and lymphatic anatomy. These technologies facilitate tailored lymphadenectomies and optimize resection strategies, ultimately improving surgical precision and patient outcomes [42,43,45].

#### 3.4.3. CRC

For CRC, diffusion-weighted MRI and PET-CT are increasingly utilized to monitor the response to neoadjuvant therapy and predict the complete pathological response. Radiomics enhances this evaluation by detecting subtle changes in tumor heterogeneity, which can inform decisions regarding organ preservation. This precise imaging-based risk stratification supports the use of more conservative surgical approaches when appropriate, thereby reducing morbidity without compromising oncological effectiveness [48].

Although AI remains a supplementary tool for clinical expertise and multidisciplinary judgment, its integration with advanced imaging and real-time decision-support systems marks a significant step toward truly individualized surgical strategies. By combining patient-specific biological and clinical parameters with sophisticated imaging analytics, this holistic approach enhances risk stratification, surgical planning, oncological outcomes, and long-term survival [48,49,70].

### 3.5. Future Perspectives

#### 3.5.1. Advances Toward Truly Personalized Care

Future advancements in gastrointestinal surgical oncology will likely focus on overcoming existing limitations to achieve personalized medical care. Although technologies such as AI and multi-omic profiling have significantly advanced personalized medicine, their integration highlights key challenges that remain unresolved.

#### 3.5.2. Data Integration and Quality

Personalized medicine relies on the integration of accurate and comprehensive data from genomic, clinical, and imaging sources. Currently, significant limitations are the variability in data quality, heterogeneity, and lack of standardized methodologies, which can affect the accuracy and applicability of personalized therapeutic strategies [5,7,49]. Establishing international standards such as Fast Healthcare Interoperability Resources (FHIR), can help harmonize diverse data types and facilitate more reliable personalized medical decision-making [71].

#### 3.5.3. Ethical and Privacy Concerns in Data Usage

The extensive use of patient-specific data, including genomic profiles and clinical records, inevitably raises critical ethical and privacy concerns. Ensuring patient confidentiality and addressing ethical issues around data sharing are essential steps toward the broader acceptance and implementation of personalized medicine [7,54]. Advanced technologies such as homomorphic encryption can securely facilitate multi-institutional data sharing without compromising patient privacy, thus addressing one of the critical unmet needs in personalized medicine implementation [54,71].

#### 3.5.4. Clinical Validation, Practical Limitations, and Trust

Although predictive models based on AI and genomic technologies offer powerful tools for personalized medicine, clinical validation remains a critical and unmet need. Personalized treatment models must undergo rigorous clinical trials and validation studies to confirm their effectiveness, reproducibility, and safety in diverse patient populations [7,49]. Achieving transparency and explainability (explainable AI) is essential for fostering clinical acceptance among healthcare providers and trust among patients [20].

Currently, significant practical limitations remain regarding the integration of AI in clinical practice. AI models trained on datasets from specific institutions may lack generalizability when applied to different patient populations or clinical environments, potentially limiting their broad clinical applicability [5,7]. Additionally, the inherent “black-box” nature of many advanced AI algorithms creates difficulty for clinicians in interpreting and trusting AI-generated results [20]. Such opacity in AI decision-making processes hampers clinical adoption and raises issues of accountability. Moreover, inaccuracies or biases in training datasets can lead to systematic errors in clinical recommendations, posing potential risks to patient safety [7,19,20,49]. Furthermore, unresolved technical, legal, and infrastructural barriers—including the cost and complexity of implementing sophisticated AI tools—remain critical obstacles to widespread adoption. Addressing these concrete and practical limitations through multidisciplinary collaboration, increased transparency, regulatory frameworks, and rigorous clinical validation is crucial for realizing the full potential of AI in gastrointestinal surgical oncology.

#### 3.5.5. Multidisciplinary Collaboration and Human Expertise

The effective implementation of personalized medicine in gastrointestinal surgical oncology relies heavily on multidisciplinary collaboration. Close coordination between surgical oncologists, radiologists, medical oncologists, geneticists, and pathologists is crucial. The role of AI and other advanced technologies should remain supportive tools integrated within existing multidisciplinary teams, complementing but never replacing expert clinical judgments [5,7,20]. Continued emphasis on human oversight, training in precision medicine principles, and education regarding the appropriate use and interpretation of advanced technologies remains essential.

In summary, the future of personalized medicine in gastrointestinal surgical oncology depends on addressing these critical limitations, enhancing clinical validation, and carefully balancing advanced technologies with clinical expertise to achieve optimal patient-centered outcomes.

## 4. Conclusions

Advances in personalized medicine, driven by AI, multi-omic profiling, and sophisticated imaging technologies, have significantly enhanced the field of gastrointestinal surgical oncology. The profound impact of technological innovation on personalized medicine is exemplified by the Human Genome Project. Initiated in 1990, this project involved international collaboration among five nations and a budget of approximately USD 3 billion. Despite significant investments, progress was initially slow, with less than half of the genome sequenced by the late 1990s. However, breakthroughs in sequencing technology have dramatically accelerated project completion, resulting in more than 50% of the genome being sequenced in the final year. Currently, sequencing an individual’s entire genome takes a few hours and costs less than USD 1000, compared with approximately 13 years and USD 3 billion during the Human Genome Project. These technological advancements continue to increase and are further strengthened by the rapid progress in AI, providing transformative developments in personalized medicine. However, critical challenges must be overcome to fully realize these clinical benefits, including standardizing data quality, addressing ethical and privacy concerns, and rigorously validating clinical efficacy. Future efforts should prioritize the development of international guidelines for data interoperability, such as the FHIR, and leveraging secure technologies, such as homomorphic encryption, to facilitate widespread, safe, and ethically sound data sharing. Moreover, continued emphasis on multidisciplinary collaboration and robust clinical validation is essential to translate these technological advances into meaningful clinical improvements. Ultimately, the successful integration of these advanced tools with human expertise promises a new era of truly personalized, patient-centered care, transforming outcomes in gastrointestinal cancer treatment.

## Data Availability

No new data were created or analyzed in this study. Data sharing is not applicable to this article.

## Abbreviations

The following abbreviations are used in this manuscript:

AI	Artificial intelligence
NGS	Next-generation sequencing
ML	Machine learning
CT	Computed tomography
MRI	Magnetic resonance imaging
CNN	Convolutional neural network
HBP	Hepatobiliary and pancreatic
DSC	Dice similarity coefficient
3D	Three-dimensional
IRCADb-1	Image Reconstruction for Comparison of Algorithm Database-1
AUC	Area under the curve
HCC	Hepatocellular carcinoma
MRCP	Magnetic resonance cholangiopancreatography
CCA	Cholangiocarcinoma
CECT	Contrast-enhanced computed tomography
GBC	Gallbladder cancer
PDAC	Pancreatic ductal adenocarcinoma
CRT	Chemoradiotherapy
PET-CT	Positron emission tomography-computed tomography
MDCT	Multidetector computed tomography
NAC	Neoadjuvant chemotherapy
pCR	Pathological complete response
NLP	Natural language processing
EHR	Electronic health record
MSI	Microsatellite instability
CI	Confidence interval
HER2	Human epidermal growth factor receptor 2
FLR	Future liver remnant
FHIR	Fast Healthcare Interoperability Resources
